# Enrichment of Neural Crest Cells by Antibody Labeling and Flow Cytometry for Single‐Cell Transcriptomics in a Lizard

**DOI:** 10.1111/ede.70030

**Published:** 2026-02-18

**Authors:** Robin Pranter, Cedric Patthey, Nathalie Feiner

**Affiliations:** ^1^ Department of Biology Lund University Lund Sweden; ^2^ Current address: Max Planck Institute for Evolutionary Biology Plön Germany; ^3^ Department of Diagnostic and Intervention Umeå University Umeå Sweden

**Keywords:** cell types, fluorescence‐activated cell sorting, HNK‐1 antibody, non‐model organism, scRNA‐seq, transcriptional profiles

## Abstract

Neural crest cells (NCCs) are a key component of the vertebrate body plan and contribute to a variety of different traits. Recent advances in single‐cell transcriptomics (scRNA‐seq) have significantly improved our understanding of NCC biology. However, their dynamic migratory behavior and spatiotemporal heterogeneity in the developing embryo pose significant challenges for their identification and isolation. Consequently, most studies of NCCs have been confined to model organisms with established transgenic tools or established methods for *in ovo* manipulation. To overcome this limitation, we present a novel approach that combines antibody labeling with fluorescence activated cell sorting to enrich for NCCs and we demonstrate the approach in the common wall lizard (*Podarcis muralis*). Through microscopy, reverse transcription quantitative polymerase chain reaction and single‐cell RNA sequencing, we show that the method enriches for NCCs as efficiently as methods relying on transgenic animals. Using this technique, we successfully characterize transcriptional profiles of NCCs in wall lizard embryos. We anticipate that this method can be applied to a wide range of vertebrates that lack transgenic tools, enabling deeper insights into the diverse roles of neural crest cells in development and evolution.

## Summary

Neural crest cells are crucial for many vertebrate adaptations. Their dynamic migratory behavior makes them challenging to study. We present an approach that isolates neural crest cells using antibody labeling and flow cytometry.

## Graphical abstract

Using wall lizard embryos, we present an approach to isolate neural crest cells by antibody labeling and flow cytometry followed by single‐cell RNA sequencing. The approach does not require transgenic animals, and thus enables addressing evo‐devo questions across a range of vertebrate taxa.

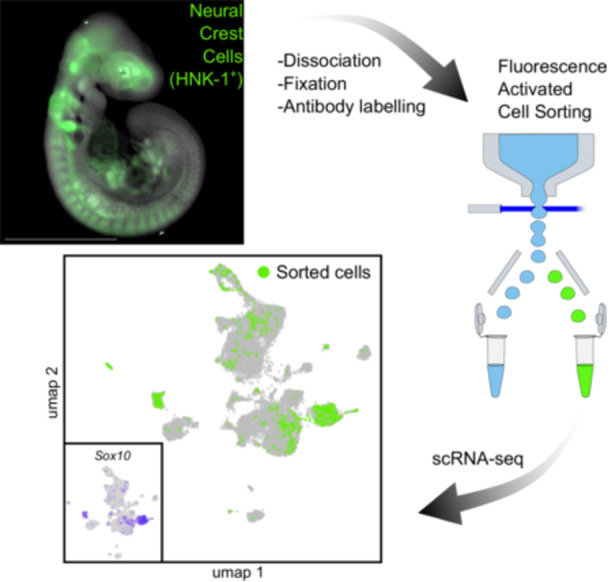

## 1 Introduction

Neural crest cells (NCCs) are multipotent stem cells of the embryo with the unique ability to generate derivatives typically associated with all three germ layers (Hall 1999; Le Douarin and Kalcheim 1999). As such, NCCs give rise to a multitude of different cell types such as chromatophores, peripheral neurons and glia, chondrocytes and osteocytes of the facial skeleton, and several types of endocrine cells such as the chromaffin cells of the adrenal gland, which are important regulators of animal behaviour. While much has been learned about the development of neural crest cells in a handful of model organisms, it remains poorly understood how the regulation of NCCs has been diversified throughout the evolution of vertebrates to give rise to variation in pigmentation, cranial morphologies and behaviours. Beyond understanding the diversification of developmental processes, from an evolutionary perspective it would be interesting to assess if the developmental linkage between certain traits imposed by their common origin in NCCs may lead to co‐evolution of these traits. For example, NCCs have been suggested to be important for the evolution of several phenotypic syndromes that have been observed in wild and domesticated animals (Brandon et al. 2023; Feiner et al. 2024; Wilkins et al. 2014).

To understand how NCCs themselves have evolved, and how they may have affected the evolution and potentially co‐evolution of NCC‐derived traits, it will be necessary to study the development of NCCs in a broad range of taxa. The current gold standard for studying cellular diversity and differentiation is single cell transcriptomics (scRNA‐seq). However, there are several challenges associated with scRNA‐seq of NCCs and their derivatives. First, NCCs are migratory and dispersed among other cells, which means that they cannot be isolated through (micro‐)dissection. Second, the ability of NCCs to differentiate into a diverse set of cell types, many resembling cells of other embryonic origins, makes it challenging to label the NCC lineage using genetic markers. Early markers will not be expressed in all derived lineages and late markers will be shared with phenotypically similar cells descending from other stem cells. To overcome these challenges, scRNA‐seq studies of NCCs have relied on established transgenic lines of mice (*Mus musculus*) and zebrafish (*Danio rerio*) that permanently express a reporter following expression of a marker gene for (a subset of) NCCs. For example, cells expressing *Sox10, Wnt1* or *Pax2* have been targeted using fluorescence activated cell sorting (FACS) (Howard et al. 2021; Soldatov et al. 2019; Xu et al. 2024). Alternatively, studies have used chicken embryos (*Gallus gallus*), which are accessible for manipulation during development, and have employed injection or electroporation of various cell‐labeling constructs such as plasmids or viruses (Jacobs‐Li et al. 2023; Morrison et al. 2017; Williams et al. 2019). However, many of the taxa that would be candidates for investigating evo‐devo questions about NCCs do not have established methods for transgenesis or other manipulations during development. Furthermore, the reproductive biology of many species may pose practical challenges, for example by restricting access to embryos to a particular time of year, necessitating methods for storing of embryos or cells. To date, many workflows in scRNA‐seq are optimized for fresh cells, and where these are not available, the only viable alternative is to resort to single nuclei RNA‐seq since isolation of nuclei is feasible from fixed or frozen tissue (Habib et al. 2017; Habib et al. 2016).

To overcome these challenges and make the isolation of NCCs available to non‐model organisms, we present a methodology based on antibody labeling of fixed cells followed by FACS and demonstrate its use in the common wall lizard (*Podarcis muralis*). This species is interesting in this context because NCCs have been implicated in the evolution of a suite of correlated traits (i.e., a phenotypic syndrome), including coloration, cranial morphology and behaviour (Feiner et al. 2024). Our previous work has characterized the dynamic distribution of NCCs in this species and found that immunohistochemistry (IHC) targeting the epitope Human Natural Killer 1 (HNK‐1) reliably marks NCCs in whole wall lizard embryos (Pranter and Feiner 2024). The labeling of NCCs by HNK‐1 was previously known in a diverse set of vertebrates such as chicken (Bronner‐Fraser 1986), four other squamate reptiles (veiled chameleon: Diaz et al. 2019; Egyptian cobra: Khannoon et al. 2021; California kingsnake: Reyes et al. 2010; brown anole: Weberling et al. 2025), and several other vertebrates (Clark et al. 2001; Erickson et al. 1989; Hirata et al. 1997; Juarez et al. 2013; Kundrát 2009; Olsson et al. 2002; Tucker et al. 1984; Vincent et al. 1983). Common wall lizards can be bred in captivity, but the breeding season is restricted to a few months per year, during which females lay 2–3 clutches with about 2–10 eggs per clutch (Böhme 1986). Thus, access to embryos is restricted to a short period, which makes the ability to store fixed cells advantageous.

**Figure 1:**
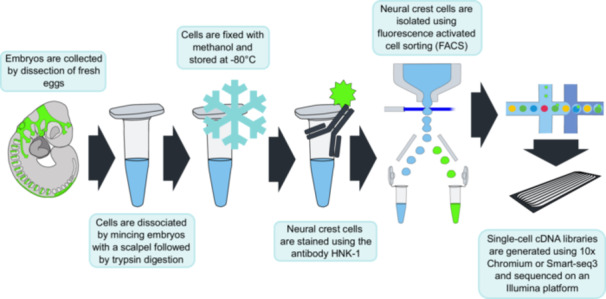
Workflow for enrichment of NCCs by HNK‐1 labeling and flow cytometry. NCCs (shown in bright green; see Pranter and Feiner 2024) and other embryonic cells (shown in grey) are dissociated into single cells, fixed and stored in methanol at −80°C. A fluorophore‐conjugated HNK‐1 antibody is used to target NCCs during flow cytometry. HNK‐1‐positive, NCC‐enriched cells are subjected to library preparation and scRNA‐seq. See Material and Methods and detailed protocol in the supplementary material. [Color figure can be viewed at wileyonlinelibrary.com]

Our methodology consists of the following steps (see Figure 1): 1) dissociation of cells from whole embryos, 2) fixation and long‐term storage of dissociated cells in methanol, 3) staining NCCs with HNK‐1, and 4) enriching for the HNK‐1‐labeled NCCs using flow cytometry. The approach is similar to previously established methods that target intracellular molecules, for example transcription factors, instead of surface molecules, to enrich for certain cell types (Hrvatin et al. 2014; Pan et al. 2011), which is commonly used in immunological studies (Albu et al. 2010; Cossarizza et al. 2017). We validate the efficiency of our protocol using a combination of microscopy, reverse transcription quantitative polymerase chain reaction (RT‐qPCR) and scRNA‐seq, and use the latter to gain insight into the transcriptional profile of NCCs in the common wall lizard.

## 2 Results

In brief, our experiments resulted in a method starting with eggs collected from a captive colony of common wall lizards that were dissected to obtain embryos. The embryos were dissociated into cell suspensions using trypsin and fixed in methanol. The dissociated and fixed cells were stored at −80°C. To enrich for NCCs, cell suspensions were stained using an HNK‐1 antibody and FAC‐sorted based on the anti‐HNK‐1 fluorescence signal. RNA was preserved during antibody staining using a MOPS‐based buffer supplemented with RNase inhibitors and DTT as previously described (Patthey et al. 2016). For a detailed protocol, see supplementary material. In the following sections we describe each step and its validation.

### 2.1 Staining NCCs With an Antibody Binding the Epitope Human Natural Killer 1 (HNK‐1)

**Figure 2:**
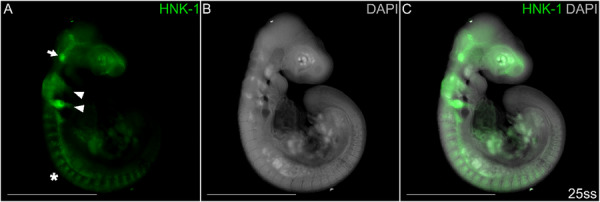
Migratory and differentiating NCCs are stained by HNK‐1 in a methanol‐fixed embryo at somite stage 25. A HNK‐1 distinctly labels cranial NCCs in migratory streams entering the second and third pharyngeal arches (arrowheads), multiple streams of migratory trunk NCCs (asterisk) and differentiating peripheral nervous system (e.g. the trigeminal nerve; arrow). B DAPI staining visualizes regions with high cellular density. C Overlay image combining HNK‐1 and DAPI signal. Scale bars are 1 mm. [Color figure can be viewed at wileyonlinelibrary.com]

NCC‐staining by the HNK‐1 antibody has previously been confirmed in *P. muralis* (Pranter and Feiner 2024), albeit with paraformaldehyde‐ and not methanol‐fixed embryos. To rule out the possibility that methanol fixation affects the HNK‐1 epitope and thereby prevents efficient labeling of NCCs fixed in methanol, we performed immunohistochemistry staining of HNK‐1 using methanol‐fixed embryos (Figure 2). The embryo was also stained with 4′,6‐diamidino‐2‐phenylindole (DAPI) to provide spatial context in the embryo. This resulted in successful labeling of NCCs, as evidenced by HNK‐1 signal in migrating NCCs entering the second and third pharyngeal arches in the head and passing through the somites in the trunk. Some HNK‐1 signal is observed in the peripheral nervous system such as the trigeminal nerve (arrow in Figure 2A), which is in agreement with previous findings (Pranter and Feiner 2024).

### 2.2 Enriching for HNK‐1‐Labeled NCCs Using Flow Cytometry

To obtain suspensions of dissociated, fixed cells, whole embryos were minced with a scalpel, further dissociated using trypsin, and filtered through a cell strainer. Dissociated cells were subjected to a fixable Live/Dead stain to label dead cells, allowing efficient identification of high‐quality cells using flow cytometry (see below). The cells in the resulting suspension were fixed using methanol and stored for up to several months at −80°C (also in methanol) prior to staining with DAPI and fluorophore (FITC) conjugated HNK‐1 antibody.

**Figure 3:**
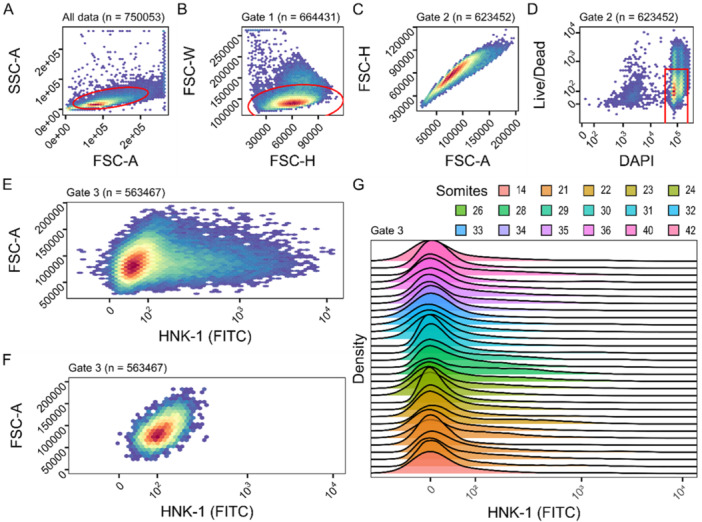
Fluorescence‐activated cell sorting of HNK‐1 positive cells. Panels A‐E show heatmaps of cell densities of one representative sample, panel F shows another sample which lacks HNK‐1 staining but is otherwise treated identically, and panel G summarizes flow cytometry results of 31 samples. The gates (subsampling strategies) are shown with red outlines (panels A, B and D). A Forward and side scatter are plotted for all cells. Gate 1 selects for proper cells and against debris. B Height and width of the forward scatter plotted for the cells in gate 1. Gate 2 selects for single cells and against doublets. C Area and height of the forward scatter confirms that no doublets are contained in gate 2. D Fluorescence from the DAPI and NIR Live/Dead stains plotted for the cells in gate 2. Gate 3 selects DAPI positive and Live/Dead negative cells, (i.e., cells with DNA that were alive before fixation). E HNK‐1 (FITC) fluorescence plotted against forward scatter area of cells in gate 3. A long tail of cells with gradually more FITC fluorescence can be seen emerging from the main cluster with low FITC fluorescence. F Same plot as shown in panel E, but for a sample that was not stained with HNK‐1 (negative control) that shows the lack of the tail of FITC‐positive cells. G The distributions of FITC signals in 31 samples. The peak of each distribution has been centered at 0 for the purpose of visualization. Overall profiles with a large peak or FITC‐negative cells followed by a long tail of gradually more FITC‐positive cells, are similar across experiments. [Color figure can be viewed at wileyonlinelibrary.com]

To select HNK‐1‐positive, high‐quality single cells, the suspensions were sorted using flow cytometry implementing a series of nested gates (i.e. sub‐setting criteria) (Figure 3). Debris and doublets were excluded based on forward‐ and side‐scatter properties (Figure 3A‐C) as well as DAPI intensity, selecting for cells with DAPI intensity characteristic of nuclei in G_1_ and G_2_ phase of the cell cycle (Supplementary figure 1). Among the selected events (corresponding to cells), variation in size and shape (forward‐ and side‐scatter) was continuous (Figure 3A‐C). The integrity of cell morphology was also confirmed by ocular examination of sorted cells using a light microscope, and no severe signs of cell leakiness were detected (Supplementary figure 2). The HNK‐1 (FITC) signal was distributed as one large cluster of FITC‐negative cells followed by a long tail of cells with gradually more FITC‐signal (Figure 3E), and samples that were not stained with HNK‐1 (FITC) lacked this tail with high FITC signal (Figure 3F). This profile was consistent across experiments (Figure 3G) and shows similarity to flow cytometry experiments sorting NCCs using GFP signal in transgenic or *in ovo* manipulated embryos (Howard et al. 2021; Jacobs‐Li et al. 2023).

### 2.3 Characterization of HNK‐1 Positive Cells Using RT‐qPCR

**Figure 4:**
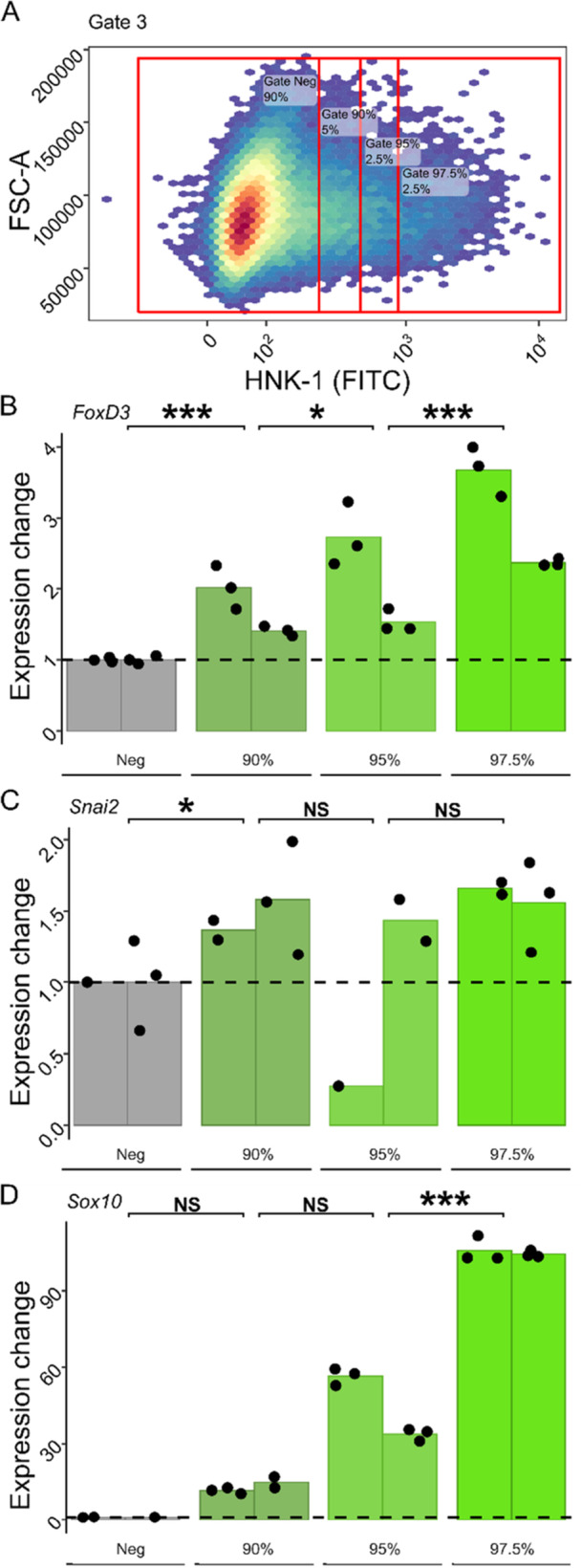
RT‐qPCR confirms gradual enrichment of NCC‐marker gene expression with increasing strength of HNK‐1 signal in flow cytometry. A Cells from one of the two pools, plotted as HNK‐1 (FITC) signal against the forward‐scatter area. The cells were further sorted using four new gates based on their FITC fluorescence (i.e., HNK‐1 signal). The first gate includes cells below the 90th percentile of the distribution (‘Gate Neg’), the second one cells between the 90th and the 95th percentiles (‘Gate 90%‘), the third one cells between the 95th and the 97.5th percentiles (‘Gate 95%’) and the fourth one cells above the 97.5th percentile ('Gate 97.5%’). (B‐D) Normalized expression divided by the ‘Gate Neg’ mean normalized expression of the NCC marker genes *FoxD3, Snai2* and *Sox10* measured by RT‐qPCR. Technical replicates are plotted as points and the mean of each biological replicate (pool) is plotted with one bar per sorting gate (error bars are omitted since data spoints are shown). Statistical significance of a positive relationship between NCC marker expression and FITC signal is indicated by asterisks. [Color figure can be viewed at wileyonlinelibrary.com]

To assess whether HNK‐1 positive cells obtained through FACS were enriched for NCCs, we collected different sets of cells from flow cytometry and subjected them to RT‐qPCR targeting the three NCC marker genes *Sox10, FoxD3* and *Snai2*, whose utility as NCC‐markers have been previously confirmed in the common wall lizard (Pranter and Feiner 2024). First, RNA integrity in fixed, frozen and sorted cells was confirmed by analysing RNA extracts using Bioanalyzer (RIN: mean = 7.7, sd = 0.99, *n* = 4) and found to be comparable to cells that were sorted directly after fixation (RIN = 6.4, *n* = 1). Then, two pools, each consisting of three embryos to ensure sufficiently high cell numbers, were stained and gated as described above and further sorted into bins with increasingly stronger HNK‐1 (FITC) signal using four additional gates (visualized in Figure 4A): the first gate included cells below the 90th percentile of the distribution (‘Gate Neg’), the second one cells between the 90th and the 95th percentiles (‘Gate 90%‘), the third one cells between the 95th and the 97.5th percentiles (‘Gate 95%’) and the fourth one cells above the 97.5th percentile ('Gate 97.5%’). RT‐qPCR of the resulting four bins showed that the relative expression level of each of the three marker genes *Sox10, FoxD3* and *Snai2* was positively correlated with the HNK‐1 (FITC) signal (Figure 4B‐D, Table 1). *Sox10* and *FoxD3* showed the highest expression levels in the 97.5th percentile bin with a marked, gradual increase from leniently to strictly sorted samples with an increasing HNK‐1 (FITC) signal. The results for *Snai2* were less pronounced, but a trend towards higher expression levels with increased HNK‐1 (FITC) signal was also evident. In summary, the RT‐qPCR results of *Sox10* and *FoxD3* strongly support an enrichment of NCCs in the fraction of cells with high HNK‐1 (FITC) signal.

**Table 1:** NCC marker expression increases with increasing HNK‐1 (FITC) signal. Resulting contrasts for linear models of relative gene expression as a function of gate averaged across biological replicates. The analysis was run as one model per marker and *P*‐values are Tukey adjusted.

Marker	Contrast	Estimate	SE	df	t ratio	*p* value
FoxD3	Neg ‐ 90%	−0.00451	0.000915	19	−4.933	0.0001
	90% − 95%	−0.00224	0.000915	19	−2.453	0.024
	95% ‐ 97.5%	−0.00677	0.000915	19	−7.403	< 0.0001
Snai2	Neg ‐ 90%	−0.000163	6.93E‐05	12	−2.344	0.0371
	90% ‐ 95%	0.000119	7.51E‐05	12	1.58	0.1402
	95% ‐ 97.5%	−0.000142	7.51E‐05	12	−1.894	0.0826
Sox10	Neg ‐ 90%	−0.00607	0.0209	15	−0.29	0.7757
	90% ‐ 95%	−0.01575	0.0174	15	−0.907	0.3789
	95% ‐ 97.5%	−0.05826	0.0165	15	−3.526	0.0031

### 2.4 Characterization of HNK‐1 Positive Cells Using scRNA‐Seq

To further validate the enrichment of NCCs using HNK‐1 labeling combined with FACS, we generated scRNA‐seq datasets from unsorted and unstained cells, cells sorted above the 85th percentile of HNK‐1 (FITC) fluorescence (leniently sorted), and cells sorted above the 95th percentile of HNK‐1 fluorescence (strictly sorted). The unsorted and the leniently sorted samples were pools of each three embryos and were subjected to the 10X Chromium protocol. The strictly sorted samples were derived from a single embryo and were subjected to the Smart‐seq3 protocol (replicated in two 384‐well plates) since this method is more suitable for samples with low numbers of cells, which result from the stricter sorting (Hagemann‐Jensen et al. 2020).

**Figure 5:**
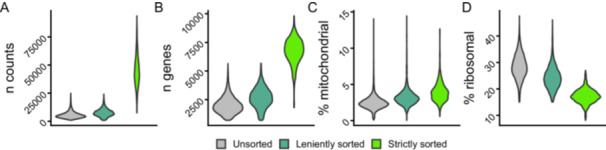
Quality metrics of the scRNA‐seq data plotted for unsorted, leniently sorted and strictly sorted cells. (10761 unsorted, 3190 leniently sorted and 704 strictly sorted cells). The values plotted on the y‐axes are: A per cell total counts of unique molecular identifiers (UMI). B per cell total gene count, C the per cell percentage of UMIs that are derived from mitochondrial genes and D the per cell percentage of UMIs that come from ribosomal genes. [Color figure can be viewed at wileyonlinelibrary.com]

Comparison between the unsorted and the leniently sorted samples (both sequenced using the same methodology) confirms that staining and FAC‐sorting does not influence standard quality metrics for scRNA‐seq data such as the gene and read counts per cell and the percentage of mitochondrial counts per cell (Figure 5, Supplementary table 1). The strictly sorted sample shows higher gene and read counts per cell, which is due to different scRNA‐seq protocols, but comparable metrics in the percentage of mitochondrial counts per cell. This is a general measure of cell stress and the range of our observed values does not indicate high stress levels (Osorio and Cai 2021). The percentage of ribosomal counts appears to decrease with stricter sorting.

Using the scRNA‐seq datasets we employed four additional tests to assess the efficiency of our approach to enrich for NCCs: 1) comparison of mean NCC‐signature scores calculated from marker gene lists between unsorted and sorted samples; 2) projection of each sample onto a reference mouse whole‐embryo atlas and qualitative assessment of cell types; 3) calculation of relative expression of the NCC marker gene *Sox10* and comparison of the percentage of *Sox10*‐expressing cells between unsorted and sorted samples of this and published datasets; and 4) shared Uniform Manifold Approximation and Projection (UMAP) embedding and comparison of the distribution of sorted cells and expression of NCC marker genes.

First, NCC enrichment was assessed by calculating an NCC‐signature score of each cell and comparing median scores between the unsorted, the leniently sorted and the strictly sorted samples. The NCC‐signature score was calculated using a core set of NCC marker genes (Simões‐Costa and Bronner 2015): *Sox10*, *FoxD3*, *Snai2*, *Pax3, Pax7, Sox9, Wnt1, Tfap2a, Sox5* and *Zic1*. The NCC‐signature score overall provided strong support for an enrichment of NCCs in the strictly sorted sample. The median NCC‐signature score for the strictly sorted sample was 0.07, while the equivalent number in leniently sorted sample was −0.010 and for the unsorted sample, it was −0.036. There were significant differences between all samples (Wilcoxon rank sum tests: all three comparisons resulted in *P*‐values <0.001).

**Figure 6:**
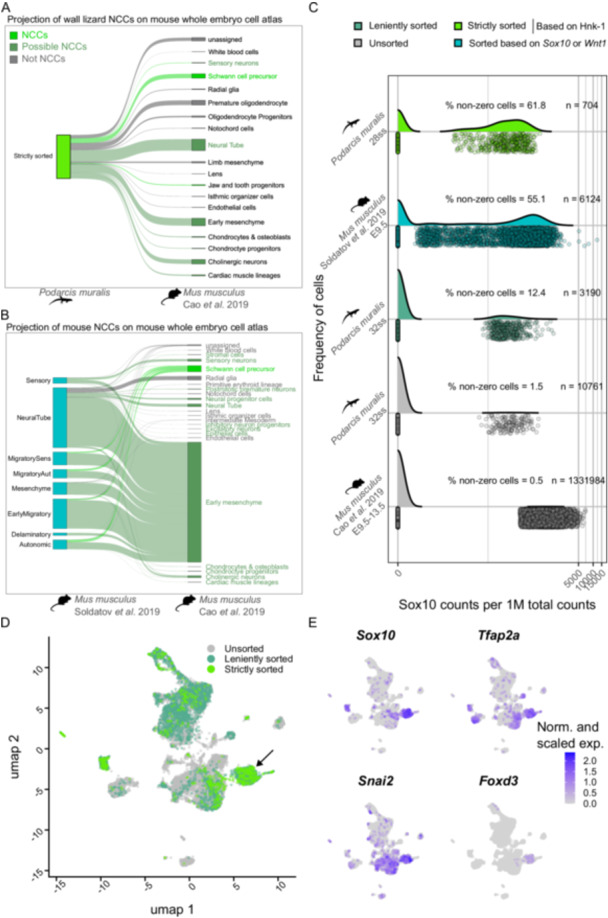
Comparison of HNK‐1‐labeled and FAC‐sorted cells of the common wall lizard to published datasets of mouse. A Projection of the strictly sorted cells onto the whole‐embryo mouse cell atlas. The projections are labeled on the right by the names of the cell atlas clusters on which they were projected, and coloured by their classification as ‘NCCs’, ‘Possible NCCs’ or ‘Not NCCs’. B For comparison, projection of a mouse NCC dataset (Soldatov et al. 2019) onto the whole‐embryo mouse cell atlas (Cao et al. 2019). The NCCs are labeled on the left by their cluster labels in the NCC dataset, and the projections are coloured and labeled on the right as in panel A. C Relative expression of *Sox10* of unsorted, leniently sorted and strictly sorted cells of the common wall lizard as well as sorted NCCs (Soldatov et al. 2019) and cells (Cao et al. 2019) from mouse. The total number of cells and the percentage of non‐zero cells are given above each curve. D All cells plotted in a UMAP and coloured by the sorting strategy. The unsorted cells occupy the largest part of the UMAP space and the strictly sorted cells are mostly restricted to a specific area of the embedding marked by an arrow. E Same plot as in D, but coloured by the expression levels of four NCC marker genes. The expression of these genes is mostly found in the same area of the UMAP where the strictly sorted cells are located (marked by arrows). Expression levels were normalized across samples to correct for sequencing depth. [Color figure can be viewed at wileyonlinelibrary.com]

Second, to classify cell types, cells from each sample were projected onto a reference dataset consisting of the mouse whole‐embryo atlas (Cao et al. 2019) using the scmap method (Kiselev et al. 2018), resulting in a cell type classification for each cell in the dataset. The cells were reclassified as ‘NCCs’ (including derivatives), ‘possible NCCs’ or ‘not NCCs’ according to established knowledge of which cell types are derived from NCCs (Barresi and Gilbert 2021; Bronner and LeDouarin 2012; Cao et al. 2019; Eames et al. 2020; Le Douarin and Kalcheim 1999). The ‘possible NCCs’ include cell types that are derived by both NCCs and other stem cells, for example chondrocytes. In the unsorted sample, 50.9% of cells were classified as either NCCs or possible NCCs, which was lower than in the mouse reference (62.1%; Cao et al. 2019). In the leniently sorted sample this percentage was 60.9% (Supplementary figure 3) and in the strictly sorted sample the corresponding value is 72.7% (the projection of the latter is shown in Figure 6A). Performing the same projection using a published dataset derived from transgenic mice that was enriched for NCCs (Soldatov et al. 2019), returned 96.1% of cells classified as NCCs or possible NCCs (Figure 6B).

Third, we assessed the efficiency of our method by calculating the per cell relative expression of *Sox10*, a canonical NCC marker gene (Simões‐Costa and Bronner 2015; Soldatov et al. 2019), as the number of counts per 1 million reads. The distribution of the relative *Sox10* expression was compared between the unsorted, the leniently sorted and the strictly sorted samples, and to two previously published datasets derived from whole mouse embryos (Cao et al. 2019) or transgenesis‐mediated, FAC‐sorted mouse NCCs (Soldatov et al. 2019). Across all datasets, the per cell relative *Sox10* expression showed a bimodal distribution with cells either expressing no *Sox10* at all, or expressing it at moderate to high levels (Figure 6C). We therefore considered it justified to compare the proportion of cells with non‐zero *Sox10* expression across datasets. Using this metric, we found that the whole‐embryo datasets were comparable between lizard (1.5%) and mouse (0.5%). The proportion of *Sox10* expressing cells was slightly increased in the leniently sorted sample with 12.4% and was considerably higher in the strictly sorted sample of the common wall lizard with 61.8%. The corresponding proportion in the FAC‐sorted mouse NCC dataset (Soldatov et al. 2019) was slightly lower with 55.1%.

Fourth, we embedded all cells in the same UMAP and compared the distribution of cells derived from unsorted, leniently sorted and strictly sorted samples to the distribution of expression levels of four NCC marker genes (*Sox10, Tfap2a, Snai2* and *FoxD3*) (Simões‐Costa and Bronner 2015). In this embedding, we find that with increasing sorting strictness, cells occupy an increasingly restricted area within the distribution of unsorted cells: leniently sorted cells take up a subspace of the unsorted cells, and strictly sorted cells are nested within a subspace of the leniently sorted cells (Figure 6D). The restricted area where most strictly sorted cells are located largely overlaps with the areas of highest expression of the four NCC marker genes (Figure 6E), indicating that the sorted cells display NCC characteristics. Considering the insights gained from the four independent tests, we conclude that our methodology using HNK‐1 labeling coupled with FAC‐sorting enriches for NCCs as efficiently as sorting based on transgenic animals.

### 2.5 Differential Expression Analysis in Unsorted Cells Reveals Putative Novel NCC Markers

**Figure 7:**
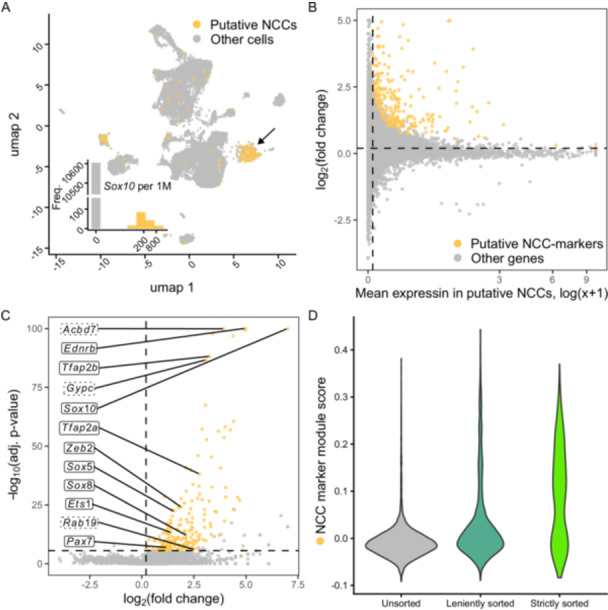
Transcriptional profile of putative NCCs and identification of putative novel NCC marker genes. A Unsorted cells plotted in the UMAP embedding constructed with all cells (same as shown in Figure 6D), coloured according to their classification as putative NCCs in orange (i.e., expressing *Sox10*) or others in grey (i.e., not expressing *Sox10*). The inset shows a histogram of *Sox10* counts per 1 million counts in all unsorted cells (equivalent to the fourth row of Figure 6C). B‐C 214 out of 20201 genes were found to be significantly more highly expressed in putative NCCs compared to other cells. Thresholds for a gene to be considered significant: average SCTransform corrected expression in putative NCCs > 0.05, average log_2_ fold change > 0.1 and Bonferroni corrected *p‐*value ≤ 0.05. In C, selected genes are labeled with their gene name (boxes with solid lines for known NCC marker genes and boxes with dashed lines for genes with previously unknown roles in NCC biology). D Distribution of NCC marker module scores based on the 214 putative NCC marker genes shown for unsorted, leniently sorted and strictly sorted cells. [Color figure can be viewed at wileyonlinelibrary.com]

By contrasting gene expression profiles between putative NCCs and other cells in the unsorted sample, we gained further insight into NCC gene expression profiles. Given the bimodal distribution of *Sox10* expression among unsorted cells (cells either express no *Sox10*, or at moderate to high levels), we defined all cells that express any *Sox10* as putative NCCs (Figure 7A). Thus, 156 out of 10605 cells (1.5%) were classified as putative NCCs. Assessing differential gene expression between these putative NCCs and all other cells identified 214 genes that were significantly higher expressed in NCCs (Figure 7B‐C, Supplementary Data Table 4). No gene was found to be significantly less highly expressed in NCCs. Among the 214 genes that were upregulated in NCCs we find several well‐known NCC marker genes such as *Pax7, Ets1, Zeb2, Tfap2a, Tfap2b, Ednrb, Sox8,* and *Sox5*, which indicates that our method recovers NCC markers previously reported for other taxa (e.g., Charney et al. 2023; Mitchell et al. 1991; Nataf et al. 1996; Schock and LaBonne 2020; Simões‐Costa and Bronner 2015). In addition, among the list of genes that are upregulated in NCCs, we also find genes whose roles in NCC biology have not been described to date, for example *Acbd7*, *Gypc,* and *Rab19*. These genes may be putative novel NCC marker genes warranting further investigation. Deriving an NCC marker module score based on the 214 genes that are upregulated in NCCs and comparing these module scores between unsorted and sorted samples reveals that the strictly sorted samples show a relatively larger proportion of cells with high NCC marker module scores (Figure 7D).

## 3 Discussion

To understand how neural crest cells have diversified across vertebrates, and how their developmental organization has shaped the evolution of NCC‐derived cells and traits, novel methods for targeting NCCs in non‐traditional model organisms are needed. To meet this demand, we developed a method for NCC isolation that does not rely on established transgenic lines. We also aimed to develop a method that can be applied to organisms that pose challenges associated with obtaining embryos and rapidly processing fresh samples on demand. To achieve this, we used the common wall lizard, in which a syndrome composed of neural crest‐derived traits has evolved (Feiner et al. 2024). We combined methanol fixation that allows long‐term storage of embryonic cells at −80°C with labeling of NCCs with the antibody HNK‐1 followed by flow cytometry. We confirmed that the resulting cells collected from embryonic common wall lizards were of high quality, contained RNA that was not degraded, and were enriched in NCCs to the same extent as published data from a transgenic mouse model.

Only a small fraction of all embryonic cells originates from the neural crest. Due to their dynamic nature and partial resemblance to cells of other embryonic origin, it is difficult to count the exact number of NCCs or neural crest‐derived cells in an embryo. However, the expression of NCC marker genes such as *Sox10* in whole‐embryo single‐cell profiling datasets that we report here suggests that NCCs make up less than 1% of all cells in an embryo. This illustrates that an enrichment strategy is necessary for a targeted study of this cell type. Our flow cytometry results showed that HNK‐1 labeled cells do not form two separated populations but rather a continuous distribution, reminiscent of results obtained using transgenic animals (Howard et al. 2021; Jacobs‐Li et al. 2023). The reason for this may be that NCCs exhibit varying levels of HNK‐1 on their surface, and even non‐NCCs, such as certain blood cells, may have HNK‐1 molecules on their surface (Abo and Balch 1981). Nevertheless, using a series of gradually stricter gating, we were able to gradually enrich our sample for NCCs. Strictly sorting samples by taking only the top ~5% of cells with the highest HNK‐1 (FITC) signal proved to satisfactorily enrich for NCCs and increased the proportion from below 1% to over 60% of isolated cells. This enrichment level is comparable to that attained by a published study using transgenic mice (Soldatov et al. 2019). When leniently sorting samples by taking the top ~15% of cells, the enrichment level dropped to below 10%, suggesting that NCCs are strongly concentrated in the positive tail of the HNK‐1 distribution.

One possible extension of the method we describe here is that different antibodies can be implemented. Using a single marker to target NCCs is not likely to capture all cells belonging to the NCC lineage. This is due to the challenging biology of NCCs with a high potency and the co‐option of cellular phenotypes otherwise expressed by cells derived from other stem cells. Since HNK‐1 mainly labels migratory NCCs, post‐migratory NCCs will likely be missed particularly at later stages of development. It appears that there is no single marker that is both *specific* enough to *only* label NCCs and their derivatives, yet *sensitive* enough to label *all* NCCs including their derivatives. This is a challenge faced both by transgenesis‐dependent and ‐independent methods. However, the flexibility of antibody‐guided FACS opens the possibility of combining different antibodies. Carefully selecting a panel of different antibodies should make it possible to enrich for a wider or more narrow range of cells and could be a further development of our methodology.

In addition to describing a new methodology, our study contributes the first unbiased list of putative NCC markers in a reptile. The list contains several genes with described functions in NCCs, for example *Ednrb* (Lee et al. 2003; Nataf et al. 1996), *Zeb2* (Charney et al. 2023; Vandewalle 2005) and *Tfap2a* and *‐b* (Mitchell et al. 1991; Rothstein and Simoes‐Costa 2020; Van Otterloo et al. 2022), and also genes that have no known function in NCC biology (e.g., *Acbd7*, *Gypc* and *Rab19*). Studies of NCCs in reptiles typically rely on marker genes described in model organisms, yet this prevents the discovery of genes that may be expressed in NCCs in only some groups of organisms. While this list of genes should be considered preliminary and non‐exhaustive due to the relatively shallow sequencing, it can be a valuable resource for further studies of NCC biology in reptiles and beyond.

In conclusion, we provide a new method for NCC‐enrichment using anti‐HNK‐1 guided flow cytometry. The value of this method is that it enables the study of NCCs using scRNA‐seq in all species in which NCCs can be labeled by HNK‐1 antibodies, and without the need for establishing transgenic tools. We anticipate that this will be important for future efforts into understanding the evo‐devo of NCCs.

## 4 Material and Methods

### 4.1 Embryo Collection

A captive colony of breeding common wall lizards, wild caught in 2018 in central Italy, was kept at Lund University (see Feiner et al. 2018a for details on housing; 2018b). Breeding groups consisted of one male and one or two females per cage, and each cage had a pot of moist sand where females laid their eggs. The eggs were collected within 24 h of oviposition. One embryo from each clutch was immediately dissected and assigned a developmental stage based on somite counts, which was taken to represent the whole clutch (Feiner et al. 2018a, 2018b). The rest of the clutch was incubated at 24°C in moist vermiculite until they reached the desired developmental stage. Somite formation has previously been found to proceed at a constant rate of four somites per day when incubated at 24°C (Feiner et al. 2018a, 2018b).

### 4.2 Immunohistochemistry of Methanol Fixed Embryos

Eggs were dissected in cold phosphate buffered saline (PBS; 10 mmol/L phosphate buffer, 2.7 mmol/L KCl, and 137 mmol/L NaCl, pH 7.4) and transferred to gradually higher concentrations of methanol in PBS containing 0.1% Tween20 and finally stored in methanol at −20°C. Whole‐mount embryos were stained using immunohistochemistry with the primary antibody HNK‐1 (1:500; Anti‐Hu CD57 eBioscience 11‐057742) following the method described in Pranter and Feiner (2024) and imaged using a Zeiss Axio Imager M2 fluorescence microscope. All reagents are listed in Supporting table 2.

### 4.3 Cell Dissociation, Fixation, and Staining

Embryos were dissected from their eggs in nuclease‐free PBS. Each embryo was photographed and then minced into fine pieces using a sterile scalpel. The minced embryo was incubated at 37°C with Trypsin (Gibco^TM^ TrypLE^TM^ Express Enzyme (1X), phenol red) to dissociate the cells into a single cell suspension. Trypsin treatment was terminated after 10 min by applying an inhibitor (Gibco^TM^ Defined Trypsin Inhibitor). The suspension was triturated 50 times up and down through a 1 mL pipet tip and then passed through a 70 μm cell strainer (Flowmi^TM^ Cell Strainer) to remove any remaining clumps. To stain the cells for viability the suspension was incubated for 12 min on ice with a Live/Dead stain (Invitrogen LIVE/DEAD^TM^ Fixable Near‐IR Dead Cell Stain Kit; L34975). Suspended cells were washed once in nuclease‐free PBS, fixed in methanol and stored in methanol at −80°C for up to several months.

The fixed cell solutions were resuspended in a permeabilization buffer (PB; 2% BSA, 100U/mL RNase‐inhibitor, 5 mM 1,4‐Dithiothreitol, 0.1 M MOPS pH 7.5, 1 mM EGTA, 2 mM MgSO4, 125 mM NaCl and 0.1% Saponin), and incubated on ice for 5 min. Once permeabilized, the cells were stained with a mix of DAPI (1:1000; ThermoScientific 62248), which stains DNA, and a FITC‐conjugated HNK‐1 antibody (1:100; Anti‐Hu CD57 eBioscience 11‐057742) in MRDB (a buffer identical to PB but lacking saponin), followed by a washing step with the same buffer and passed through a 70 μm cell strainer.

### 4.4 Flow Cytometry and FACS

Dissociated cells were sorted in a FACS ARIA III machine. Debris, doublets and cells that were dead before fixation were removed by three gates (Figure 3A‐D). The first gate excluded debris by excluding cells with low values in both forward and side scatter area (Figure 3A). The second gate removed doublets by excluding cells with high values in forward scatter width, which can be clearly made out when forward scatter width and height are plotted against each other (Figure 3B). The third gate further removed additional (large) debris, non‐nucleated cells and cells that were dead before fixation by only selecting cells with high DAPI fluorescence and low live/dead fluorescence (Figure 3D). The remaining cells were inspected and sorted based on their anti‐HNK‐1 (FITC) signal (Figure 3E).

### 4.5 RT‐qPCR

Cells were dissociated, fixed and stained as described above and then FAC‐sorted into 200 µL of MRD buffer (identical to PB but lacking saponin and BSA) following the gating described above. An appropriate volume was moved to a new tube based on the known concentration of cells. Total RNA was extracted using the RNeasy Micro Kit including DNase digestion on the membrane and stored in nuclease‐free water at −80°C. RNA was reverse transcribed into cDNA using SuperScript III Reverse Transcriptase and oligo‐dT primers according to the manufacturer's instructions. The resulting cDNA was used as template for qPCR of *FoxD3*, *Snai2* and *Sox10*. The gene *GAPDH*, which has an expected constant expression, was analysed together with the three NCC‐markers and was used as a reference in the normalization procedure. See Supporting table 3 for primer sequences. Technical replicates were filtered based on their melting‐curves to exclude incorrect amplification such as primer dimers, and the resulting Cq values were normalized as:

$$Cq_{\mathrm{norm}}=2^{\left(Cq_{\mathrm{GAPDH}}-{\mathrm{Cq}}_{\mathrm{marker}}\right)}$$

Differences in normalized Cq values between samples collected from different FACS gates were modelled linearly with normalized Cq as the dependent and biological replicate and FACS gate as factors. Least square means were estimated pairwise between each gate and its immediate neighbour(s) and *p*‐values were Tukey corrected using the ‘emmeans’ package in R (Lenth 2024).

### 4.6 Single Cell Transcriptomic Analysis

#### 4.6.1 10X Chromium

We used the 10X Chromium protocol for unsorted and leniently sorted samples. Cell‐dissociations from 12 embryos with the average somite stage 32 were grouped into four pools. Two pools were put together from unsorted cells directly from −80°C storage. Two pools were put together from FAC‐sorted samples following the staining and gating described above with a final sorting gate taking the cells above the ~85th percentile of the FITC distribution. The collected cells were pelleted by centrifugation, resuspended in methanol and stored at −80°C for 4 days before cell capture, and library preparation at the Eukaryotic Single Cell Genomics Facility, which is part of SciLifeLab Sweden. The pools were processed into cDNA using 10X Chromium Next GEM Single Cell 3' Reagent Kits v3.1 (Dual Index) and sequenced on an Illumina NovaSeq 6000. The target was ~5000 cells per sample and ~20k read pairs per cell. Reads were mapped to the PodMur_1.0 reference genome (Andrade et al. 2019) using the annotation GCF_004329235.1 and gene by cell count matrices were generated using the Cellranger 9.0.0 pipeline from 10X Genomics (Zheng et al. 2017) and further analysed using the R package Seurat 5.2.1 (Hao et al. 2024).

The count matrix was filtered in several steps. First, genes with fewer than 4 UMIs in the dataset, and cells that had less than 700 expressed genes, were removed. Second, cells whose transcriptome consisted of more than 15% mitochondrial reads were removed to exclude leaky cells and cells with less than 15% ribosomal reads were removed to exclude outliers. Doublets were predicted using DoubletFinder V2.0 (McGinnis et al. 2019) and removed from the dataset. Lastly, cells with more than 25,000 reads were removed to exclude outliers. Expression levels were normalized using SCTransform (Choudhary and Satija 2022; Hafemeister and Satija 2019) and variation associated with the percentage of mitochondrial reads and cell cycle state were removed by regression. The latter was modelled using the expression of orthologs of human markers for either the S‐phase (38 genes) or the G‐ and M‐phases (37 genes) (Tirosh et al. 2016). After filtering and normalization, the read counts, gene counts, percentages mitochondrial counts, percentages ribosomal counts and cell cycle scores did not appear to associate with the distribution of cells in reduced dimension space (PCA, tSNE and UMAP) or differ between predicted clusters.

#### 4.6.2 Smart‐seq3

Because cell counts in flow cytometry indicated that the number of cells obtained through strict sorting would be too few to allow application of the 10X Chromium method (in particular for early embryos), we used the Smart‐seq3 protocol for strictly sorted samples. A cell dissociation from a 28ss embryo was stained and FAC‐sorted as described above with a final sorting gate taking the cells above the ~95th percentile. The cells were sorted directly onto two 384 well plates using the ‘single‐cell’ mode on the same FACS Aria III as described above. Plates were submitted for library preparation and sequencing to the Eukaryotic Single Cell Genomics Facility at SciLifeLab in Solna, Sweden. cDNA was prepared in each well following the Smart‐seq3 protocol (Hagemann‐Jensen et al. 2020) and sequenced on an Illumina NovaSeq X Plus. Reads were mapped to the PodMur_1.0 reference genome (Andrade et al. 2019) and the count matrices were generated using the zUMIs pipeline (Parekh et al. 2018). Count matrices were further analysed using the Seurat 5.2.1 package in R (Hao et al. 2024). The count matrix was filtered to exclude cells with less than 1000 expressed genes, more than 8% mitochondrial reads and/or less than 86% exonic reads. The two filtered count matrices were normalized using the SCTransform function in Seurat (Choudhary and Satija 2022; Hafemeister and Satija 2019) and variation associated with percentage mitochondrial reads, and cell cycle state was regressed out. After filtering and normalization, the read counts, gene counts, percentages mitochondrial counts, percentages ribosomal counts and cell cycle scores did not appear to associate with the distribution of cells in reduced dimension space (PCA, tSNE and UMAP) or differ between predicted clusters.

#### 4.6.3 Assessment of NCC Enrichment

We employed four different approaches to assess the efficiency of the enrichment for NCCs, and describe each of them here in turn.

To derive a NCC‐signature score per cell, we used the function AddModuleScore in the Seurat package with the following gene set: *Pax3, Pax7, FoxD3, Sox10, Sox9, Wnt1, Tfap2a, Sox5, Snai2* and *Zic1* (Simões‐Costa and Bronner 2015). We then calculated the NCC‐signature scores for all cells in a given dataset and compared their mean across datasets.

To assess the proportion of cells expressing a core NCC marker gene, *Sox10*, we calculated the proportion of cells with non‐zero expression of *Sox10* from each filtered count matrix and from the count matrix of published datasets from mouse (Cao et al. 2019; Soldatov et al. 2019) using this formula:

$$E_{\mathrm{gene}}=\frac{C_{\mathrm{Sox}10}}{\frac{C_{\mathrm{total}\;\mathrm{RNA}}}{10^{6}}}$$

Where $C_{\mathrm{Sox}10}$ is the number of reads assigned to *Sox10* per cell, $C_{\mathrm{total RNA}}$ is the total read count per cell, and $E_{\mathrm{gene}}$ is the relative expression of *Sox10* per cell. From this, the proportion of cells with non‐zero *Sox10* expression was derived for each sample.

To derive a classification of cells into cell types, the count matrices were independently projected onto a reference mouse whole‐embryo cell atlas (Cao et al. 2019) using the scmapCluster() function in the scmap 1.28 package (Kiselev et al. 2018) using 1500 variable features and a likelihood threshold of 0.03. Gene orthology was assigned using an orthology table downloaded from Ensembl and only one‐to‐one orthologues were retained. For comparison, a dataset with sorted NCCs from mouse embryos (Soldatov et al. 2019) was also projected using the same likelihood threshold and number of variable features.

To embed all cells in a common dimensionality reduction, the 10X Chromium data and the Smart‐seq3 data were merged. To correct for batch effects, the data was first normalized separately sample by sample using SCTransform. Principal components were calculated from the normalized data. The samples were then integrated using the ‘harmony’ algorithm (Korsunsky et al. 2019) as implemented in the IntegrateLayers function in Seurat. The batch corrected data was then visualized in a UMAP.

#### 4.6.4 Identification of Putative NCC Marker Genes

By investigating the unsorted cells closer we derived a list of putative NCC marker genes. *Sox10* expressing cells were selected as ‘Putative NCCs’ and *Sox10* negative cells as ‘Other cells’. Putative NCC marker genes were derived from a differential gene expression analysis contrasting putative NCCs and other cells using the Seurat function FindMarkers. The following criteria were applied for calling differentially expressed genes: average normalized expression > 0.05, log_2_ fold change > 0.1 and Bonferroni corrected *p*‐value < 0.05.

## Conflicts of Interest

The authors declare no conflicts of interest.

## Supporting information


**Supplementary File.** Supplementary information including supplementary Figures 1‐3, supplementary Tables 1‐3, and a detailed lab protocol of the method outlined in the paper.

**Supplementary Table 4.** Information on the 19855 genes analyzed for differential expression between putitive NCCs and other cells from the unsorted samples.

## Data Availability

All sequence data generated in this study have been deposited in NCBI GEO with accession number GSE319069. Code to reproduce the results presented in this study are deposited at Zenodo together with qPCR and flow cytometry data DOI:10.5281/zenodo.18544211.
